# Visual analysis of ovarian cancer immunotherapy: a bibliometric analysis from 2010 to 2025

**DOI:** 10.3389/fmed.2025.1573512

**Published:** 2025-07-11

**Authors:** Yingjie Zhang, Yanyan Chen, Chunru Chen, Xiaohua Cheng, Yanan Peng, Juan Wang, Fuxia Li, Li Wenting

**Affiliations:** First Affiliated Hospital, School of Medicine, Shihezi University, Shihezi, China

**Keywords:** bibliometric, CiteSpace, hotspot, immunotherapy, VOSviewer ovarian Cancer

## Abstract

Research on immunotherapy for ovarian cancer is rapidly advancing, and harnessing the immune system to fight tumors is at the forefront of cancer treatment. This article aims to discuss the prospect and development trend of immunotherapy for ovarian cancer from the perspective of bibliometrics. Articles about tumor burden and immunotherapy were collected from the Web of Science Core Collection (WoSCC) (retrieved on 1 May 2025). R package “Bibliometrics” analyzes key bibliometric characteristics and creates a three-filed map to show the relationships between institutions, countries, and keywords. VOSviewer is used for co-author analysis, co-occurrence analysis, and visualization. CiteSpace calculates citation burst citations and keywords. A total of 1,449 publications were retrieved from 15 years of scientific research. The China and United States (US) published the most articles. The most productive journals were Cancer Immunology Immunotherapy and Journal for Immunotherapy of Cancer. The top institution with the highest output was HARVARD UNIVERSITY. In recent years, the hot keywords of strong citation burst strength were “dendritic cells,” “monoclonal antibody,” and “adoptive immunotherapy.” This bibliometric analysis mapped a basic knowledge structure. The tumor burden and immunotherapy field is entering a rapidly growing stage and keeping its value for future research.

## Introduction

Ovarian cancer remains one of the most prevalent and lethal malignancies in the female reproductive system. Global incidence has risen from 159,096 to 298,876 cases from 1990 to 2021, while the age-standardized incidence rate (ASIR) decreased from 7.22 to 6.71 (EAPC: −0.38%). Mortality increased from 100,584 to 185,609, yet the age-standardized mortality rate (ASDR) fell from 4.73 to 4.06 (1). The absence of overt early symptoms and effective screening methods results in approximately 70% of patients being diagnosed at advanced stages (III or IV). Survival rates vary widely (30–50%) due to high recurrence and chemotherapy resistance. Despite advances in immunotherapy and targeted treatments, the overall survival rate remains unsatisfactory, highlighting the need for further research and innovative strategies.

In recent years, immunotherapy has emerged as a transformative approach in oncology, leveraging the body’s immune system to combat malignancies. For ovarian cancer, immunotherapy holds considerable potential, encompassing strategies such as immune checkpoint inhibitors (2–4) (e.g., anti-PD-1/PD-L1 and anti-CTLA-4 antibodies), chimeric antigen receptor (5) (CAR) T-cell therapies, cancer vaccines (6, 7), and oncolytic virotherapy (8). These approaches aim to stimulate the patient’s immune response, reprogram the immunosuppressive tumor microenvironment, and enhance antitumor efficacy. Nevertheless, the complex tumor microenvironment, immune escape mechanisms, and significant heterogeneity among patients in ovarian cancer contribute to the fact that the efficacy of immunotherapy varies from one patient to another.

With the continuous expansion of research on immunotherapy for ovarian cancer, the number of relevant academic publications has increased rapidly, which reflects the evolution of research trends and focuses in this field. Bibliometrics, as a quantitative analysis method, provides valuable insights into scientific output by identifying highly influential publications, research collaborations, and emerging topics (9). Although bibliometrics studies have been widely applied in immunotherapy, systematic bibliometrics analyses specifically targeting immunotherapy for ovarian cancer still remain inadequately explored.

This study utilized the bibliometric method to systematically elaborate on the research status and development trends in ovarian cancer immunotherapy. Through in-depth analysis of the influential countries, institutions, authors, and journals within this field and the identification of high-impact keywords and emerging research topics (10). This paper aims to provide a comprehensive overview of this field and offer beneficial information for future research.

## Methods

### Data source and search strategy

Web of Science (WoS) is recognized as one of the most comprehensive academic databases worldwide, encompassing approximately 14,090 scientific journals (11, 12). For this reason, it was chosen as the primary database for this study. A comprehensive literature search was performed on May 1, 2025, focusing on studies published since 2010. Relevant records were retrieved from the Web of Science Core Collection (WoSCC) database. The search strategy was defined as follows: [Topic Search] = (Ovarian Cancer) AND (immunotherapy OR immunotherapies OR immunotherapeutic OR immunotherapeutics). The search was restricted to publications classified as articles, excluding retracted publications, retractions, and book chapters. The language filter was set to English (13). For further analysis, selected records were exported in plain text format, including complete bibliographic details and cited references.

### Software tools and respective functions

The software tools used for bibliometric analysis included the Bibliometrix R package (14), VOSviewer (15), and CiteSpace (16). Bibliometrics, primarily designed for quantitative analysis, utilized extraction methods such as authors (AU field), institutions (AU_UN field), countries (AU_CO field), publication year (PY field), keywords (DE field), and citations (TC field). This review, Bibliometrix version 4.0.0 was used to count publications and citations, measure keyword frequency, assess collaboration strength among countries and authors, and create a three-field plot for keyword analysis.

**Figure 1:**
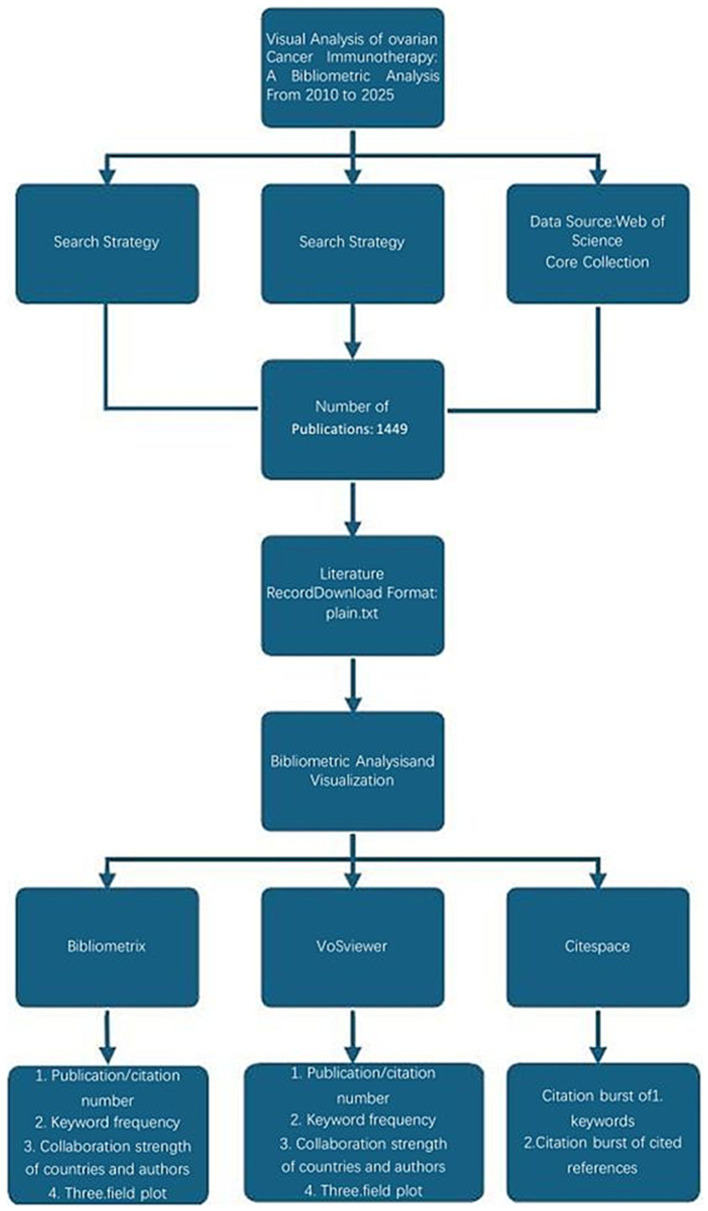
Workflow of the study.

VOSviewer, a powerful tool for co-authorship and co-occurrence analysis, uses an embedded clustering algorithm (17, 18). This study helped illustrate collaboration among authors and institutions and the relationships among keywords, with a time-overlay feature for tracking trends (19, 20). CiteSpace, specialized in citation analysis and visualization, was employed to map scientific knowledge by identifying highly cited references and keywords with strong citation bursts over time. Additionally, the online bibliometric platform^1^ was used to visualize international collaborations. An overview of the bibliometric process is shown in Figure 1.

## Results

### Analysis of annual publication output

**Figure 2:**
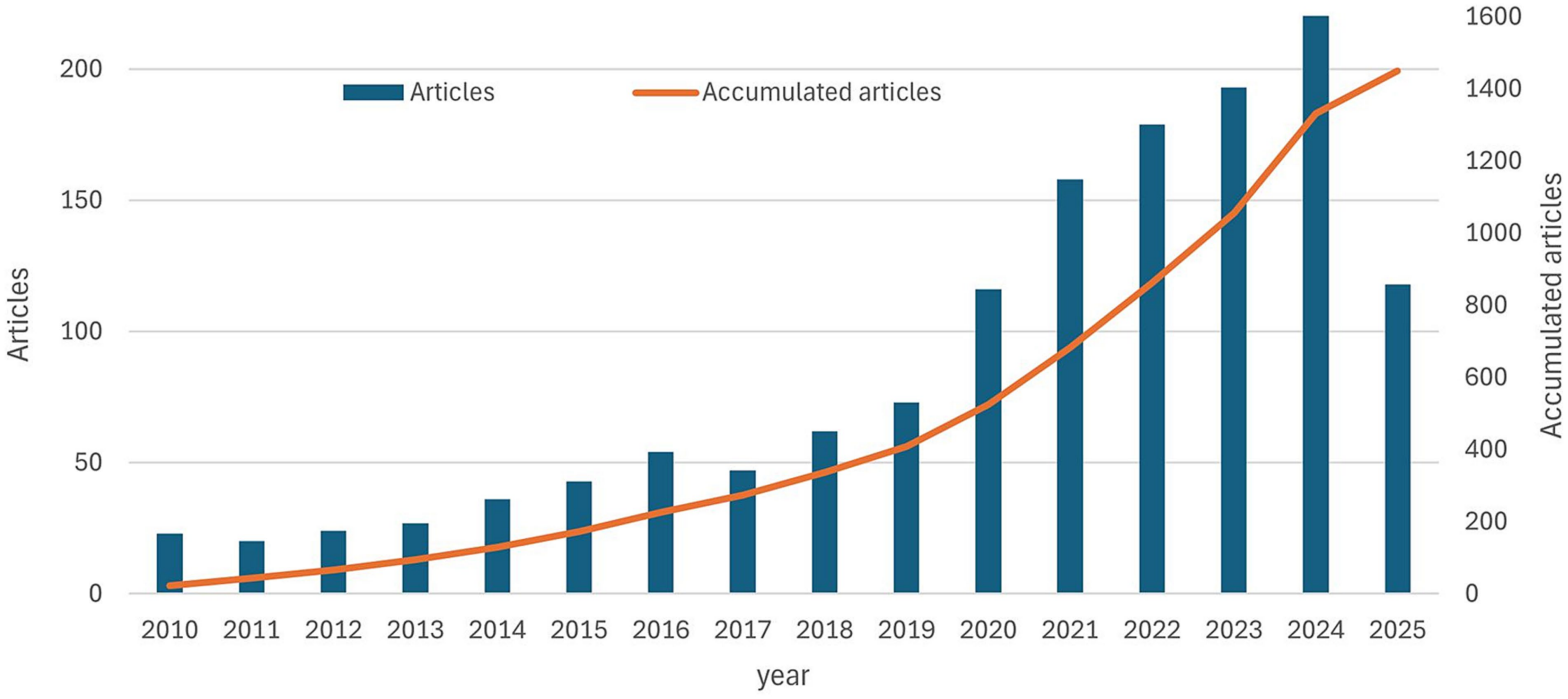
The annual number and the cumulative number of publications.

From 2010 to May 1, 2025, 1,449 publications on ovarian cancer immunotherapy were retrieved, spanning 15 years. Figure 2 illustrates the annual and cumulative number of articles on this topic. A growth rate of 11.52% was observed. From 2010 to 2019, the cumulative number of publications steadily increased from 23 to 409. In the subsequent 5 years, from 2019 to 2024, there was a rapid surge in publication output, with the cumulative total reaching 1,449 by May 1, 2025.

### Analysis of national publication volume and collaboration

**Figure 3:**
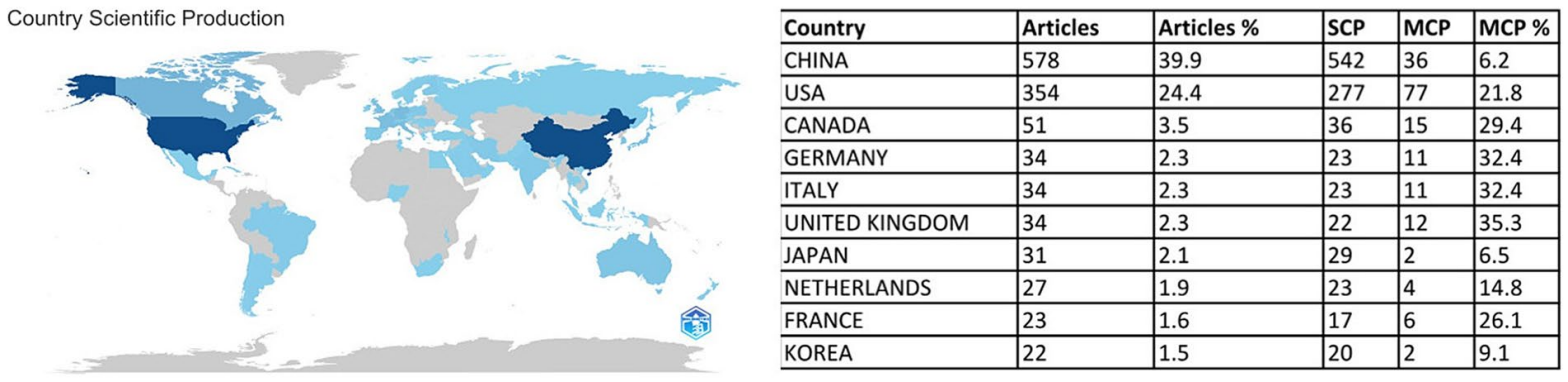
A map of country contribution based on the article’s output.

After analyzing the global publication output, it was found that 56 countries or regions have contributed to ovarian cancer immunotherapy. Figure 3 clearly illustrates the distribution of publications by country. China leads with 578 articles, accounting for 39.9% of the total, highlighting its significant contribution to this area. The United States follows with 354 articles, representing 24.4%, while Canada ranks third with 51 articles, making up 3.5%. Germany published 34 articles, accounting for 2.3%. Among the top 10 countries, Netherlands, France and Korea had fewer than 30 publications.

The Multiple Country Publications (MCP) metric effectively reflects the extent of international collaboration. While the United States leads in MCP value with 77, its MCP ratio—calculated as the ratio of MCPs to total publications—is only 24.4%, indicating a moderate share of collaborative articles. To gain further insights into the collaboration trends among countries, Supplementary Figure 1 provides a detailed analysis. The most frequent collaboration path is between the United States and China, with 42 joint publications. The United States also collaborates frequently with Canada (21 collaborations), Germany (or the UK) with 21, and Italy with 14, among the top 10 international collaboration relationships, all but the collaborations between Italy and the UK, China and Germany, and Germany and Italy, United States and Poland, underscoring the central role of the United States in global scientific collaboration.

In the table of Citing figures from national publications (Supplementary Table 2) China leads in SCI paper output, publishing 578 (39.9%) compared to the US’s 354 (24.4%), yet lags significantly in average citations per paper (8.9 vs. 34.0, 26%). This disparity reflects China’s dependence on domestic research, with only 6.2% of its papers being international collaborations (vs 21.8% in the US), despite MCPs being crucial for global impact. Structural issues include low-quality papers dominating China’s output (non-SCP average <3 vs. 24.6 in the US) and minimal international collaboration depth. Comparisons with high-performing nations like Japan (quality-focused) and the Netherlands (emerging MCP potential) suggest China must balance scale with sophistication—enhancing international cooperation, enforcing stricter quality standards, and deepening research integration—to transform from a publication leader to an innovation hub.

### Analysis of institutional output and collaboration

**Figure 4:**
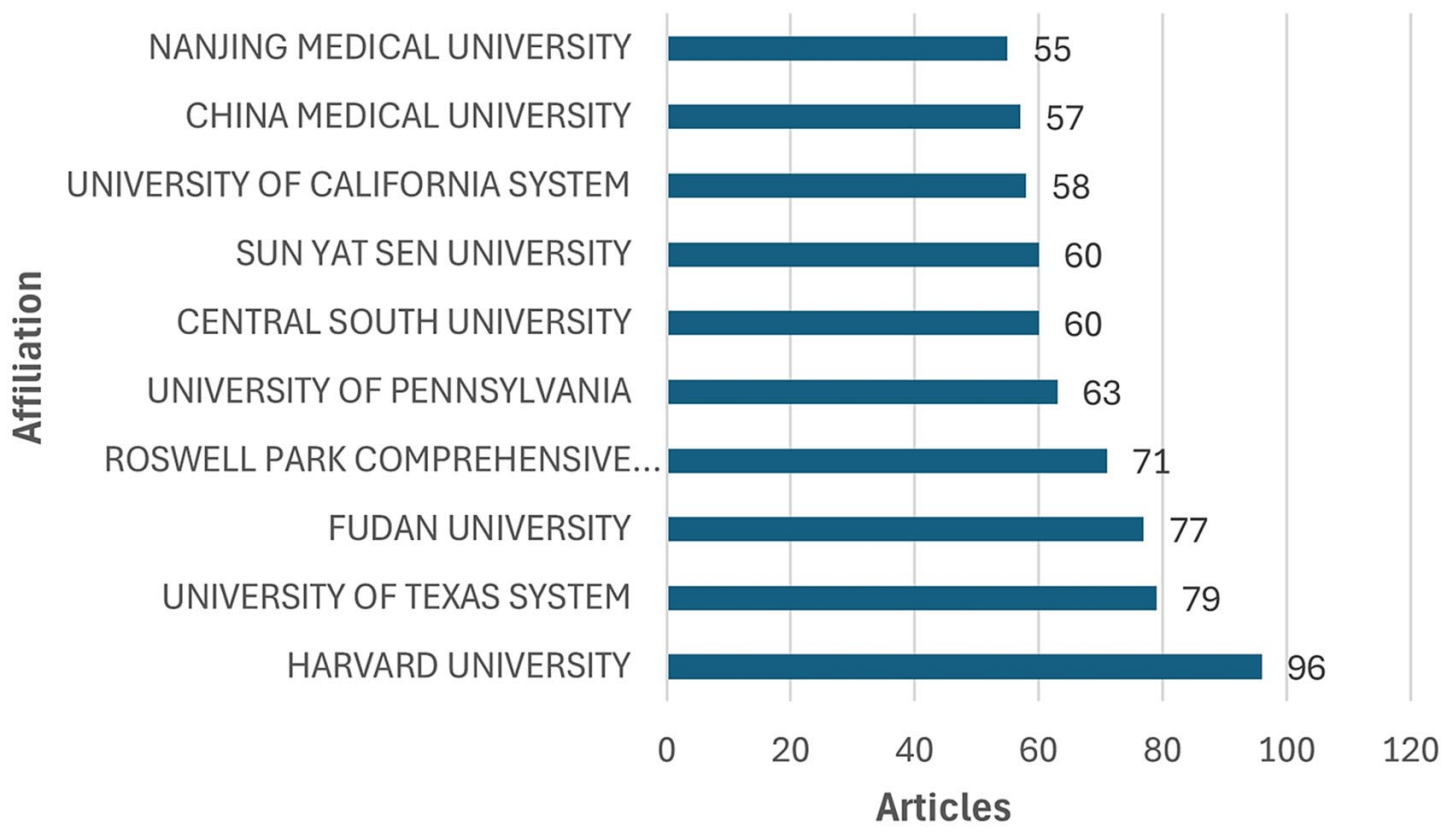
The top 10 institutions with the most publications.

In total, 1,577 institutions have conducted research related to ovarian cancer immunotherapy. The top 10 institutions are listed in Figure 4. Among these, five are based in China, and five are in the United States. Harvard University ranks first, with the highest publication output of 96 articles.

Additionally, a co-authorship analysis was performed to investigate collaboration patterns among institutions. In the clustering network generated through co-authorship analysis (Supplementary Figure 2A; Supplemental Digital Content 1), the node size reflects the number of articles published by each institution. In contrast, node color represents the clusters identified by computational classification based on collaboration intensity. The network revealed that 30 institutions are distributed across six clusters, with the red cluster containing the highest number of institutions, containing seven institutions.

In the co-authorship clustering network with a time-overlay analysis (Supplementary Figure 2B), colors represent the average publication activity of institutions in tumor burden and immunotherapy over time. Supplementary Figure 2B shows that institutions such as the University of Pennsylvania and the University of Chicago were early pioneers in this research area. In contrast, researchers from Fudan University and Sun Yat-sen University in China have made significant advancements in ovarian cancer immunotherapy in recent years. This analysis highlights the temporal progression of institutional contributions, providing insights into evolving research trends in this field. These findings provide valuable insights into institutional contributions and collaboration in ovarian cancer immunotherapy research.

### Analysis of article output and impact of journals

Among the 1,449 articles retrieved in this study, 338 journals contributed to their publication. Table 1 lists the top 10 journals ranked by article output and their most recent impact factors. The Swiss-based journal Cancers ranks first, with a total of 73 articles, closely followed by Frontiers in Immunology, reflecting comparable impact. Seven of these top 10 journals are classified within the first quartile (Q1) of the Journal Citation Reports (JCR), indicating their high quality and influence.

**Table 1:** Top 10 journals with the most articles about ovarian cancer immunotherapy.

Rank	Journals	Articles	Country	IF	JCR-c
1	Frontiers in Immunology	88	Switzerland	5.7	Q1
2	Cancers	78	Switzerland	4.5	Q1
3	Gynecologic Oncology	43	US	4.5	Q1
4	Frontiers in Oncology	37	Switzerland	3.5	Q2
5	Journal of Ovarian Research	36	UK	3.8	Q1
6	Cancer Immunology Immunotherapy	35	US	4.6	Q1
7	Journal for Immunotherapy of Cancer	35	US	10.3	Q1
8	International Journal of Molecular Sciences	31	Switzerland	4.83	Q1
9	Oncoimmunology	24	US	6.5	Q2
10	Clinical Cancer Research	19	US	10.4	Q1

Regarding publishers, five journals are based in Switzerland, five in the United States. These findings underscore the diversity of publication platforms contributing to disseminating research on ovarian cancer immunotherapy.

### Analysis of author influence and collaboration

A total of 7,375 authors have contributed to research on ovarian cancer immunotherapy. Table 2 lists the most influential authors in this field. The most authoritative author is ODUNSI K, with 20 publications and an H-index of 14. The second most influential author is COUKOS, with 15 publications and an H-index of 13, followed by KANDALAFT, who has 14 publications and an H-index of 12.

**Table 2:** Top 10 authors with the most influential about ovarian Cancer Immunotherapy.

Author	Publication	h_index	g_index	m_index	TC	NP	PY_start
Odunsi Kunle	20	14	19	1.167	766	19	2013
Coukos George	15	13	14	0.929	1067	14	2011
Kandalaft Lana E.	14	12	12	0.857	1017	12	2011
Nelson Brad H.	17	12	17	1.091	1133	17	2014
Powell Daniel J. JR.	9	9	9	0.643	584	9	2011
Nijman Hans w.	11	8	10	0.533	216	10	2010
Arend Rebecca C.	7	7	7	0.778	160	7	2016
Coleman Robert L.	11	7	10	0.583	221	10	2013
Conejo-Garcia Jose R.	8	7	8	0.467	632	8	2010
Coosemans AN	10	7	10	0.583	122	10	2013

Supplementary Figure 3A (Supplemental Digital Content 1) presents a co-authorship clustering network, revealing the collaboration relationships among researchers. The node size reflects the number of publications by each author, while the color indicates the strength of collaboration within each cluster. A total of 50 authors are divided into 13 clusters, with red, yellow, orange, and green clusters showing inter-collaboration. Supplementary Figure 3B visualizes the time-overlay analysis of co-authorship for 30 researchers. Notably, researchers Cheng Shanshan and Wang Chao are actively researching ovarian cancer immunotherapy, as evidenced by their ongoing collaborations and publication output.

### Research hotspots

#### Most cited publications

The most cited publications in a specific field highlight the research’s impact and influence. Supplementary Table 1 lists the top 10 most cited publications on ovarian cancer immunotherapy published between 2010 and 2025. The table shows that 50% of these publications have been cited over 400 times. The most frequently cited article is “Epithelial Ovarian Cancer: Evolution of Management in the Era of Precision Medicine (21)“published in 2020 in CA: A Cancer Journal for Clinicians. The second most cited publication is titled “Safety and Antitumor Activity of Anti–PD-1 Antibody, Nivolumab, in Patients With Platinum-Resistant Ovarian Cancer (21),” published in 2015 in the Journal of Clinical Oncology.

#### Citation burst analysis of references

In Supplementary Figure 4, the top 25 most-cited publications are presented. The dark blue lines represent the citation burst periods from 2010 to 2025, with a minimum burst duration of 1 years. The highest citation burst value is associated with the article “Safety and Antitumor Activity of Anti–PD-1 Antibody, Nivolumab, in Patients With Platinum-Resistant Ovarian Cancer (22)“by Hamanishi et al., which had a citation burst of 40.46 from 2016 to 2020. The second and third most-cited articles are by Julie R. Brahmer, titled “Safety and Activity of Anti–PD-L1 Antibody in Patients with Advanced Cancer (23)“(citation burst = 15.89), and by Wei-Ting Hwang, titled “Prognostic Significance of Tumor-Infiltrating T Cells in Ovarian Cancer: A Meta-Analysis” (citation burst = 15.89).

Additionally, from 2020 to 2025, three publications consistently saw increased citations, with a citation burst of 12.78. One notable article is “Ovarian Cancer Immunotherapy and Personalized Medicine (24).” The second most popular publication in recent years is “Immunotherapy for Ovarian Cancer: Adjuvant, Combination, and Neoadjuvant Therapies (25).”

#### Keyword occurrence and co-occurrence analysis

**Figure 5:**
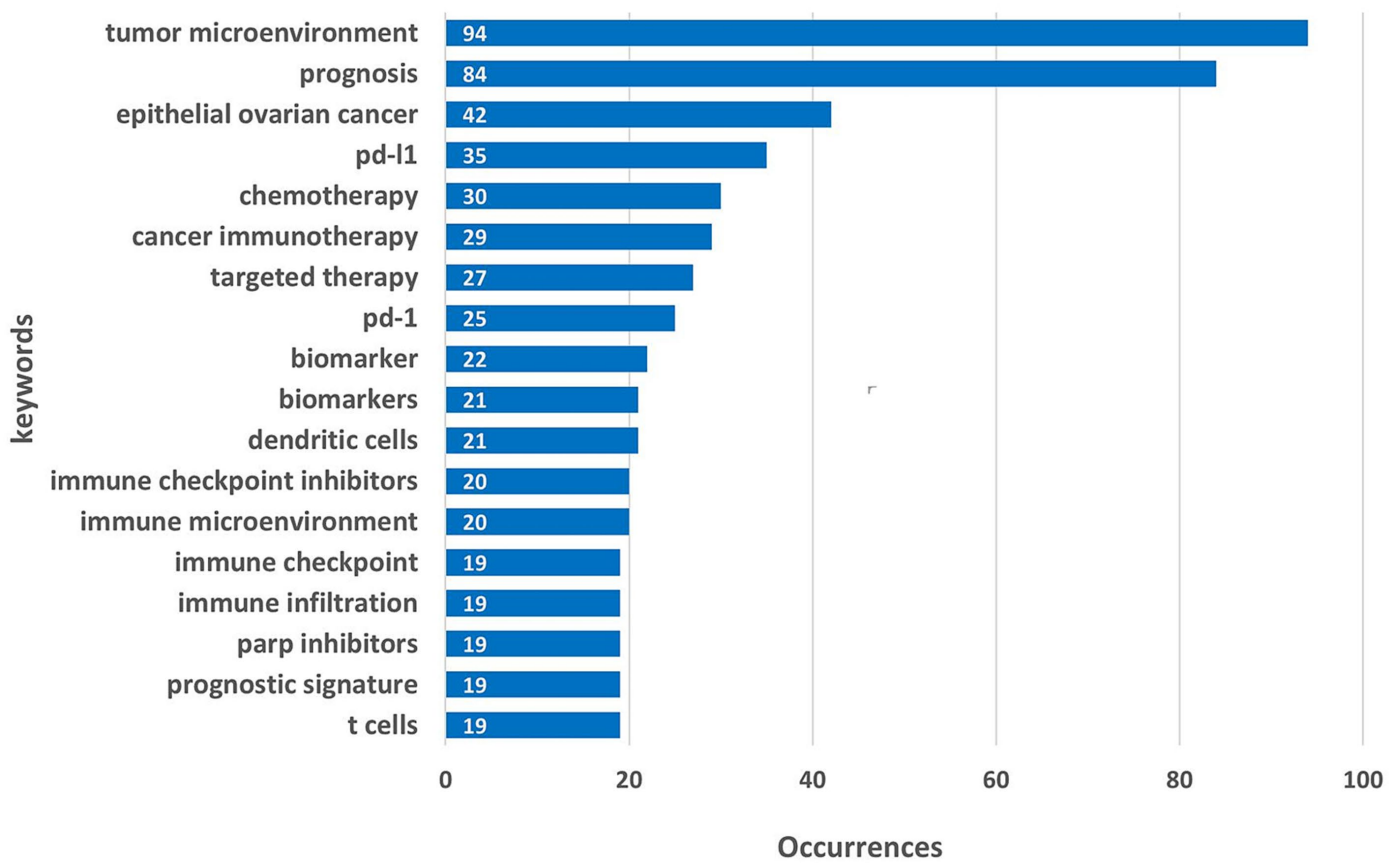
The top 20 most-used keywords (eliminated ovarian cancer and immunotherapy).

In this study, 2,267 keywords were collected, and Figure 5 presents the top 20 keywords ranked by frequency. The most frequent keyword was “tumor microenvironment,” which appeared 94 times, followed by “prognosis” (84 occurrences) and “epithelial ovarian cancer” (42 occurrences). Among the top 20 keywords, cancer-related terms include “epithelial ovarian” (42occurrences). The most frequently mentioned. Keywords related to the tumor microenvironment, such as “tumor microenvironment” (94 occurrences), “immune microenvironment” (20 occurrences), and “immune infiltration” (19 occurrences), suggest that researchers are paying increasing attention to the interaction between the tumor and its surrounding microenvironment. The relatively high frequency of treatment - related keywords, including “chemotherapy” (30 occurrences), “targeted therapy” (27 occurrences), “PD - 1” (25 occurrences), “PD - L1” (35 occurrences), “immune checkpoint inhibitor” (20 occurrences), “PARP inhibitor” (19 occurrences), implies the continuous exploration of various treatment strategies for ovarian cancer. Biomarkers / “biomarker” (22 occurrences) are also crucial for diagnosis, treatment selection, and prognosis prediction.

**Figure 6:**
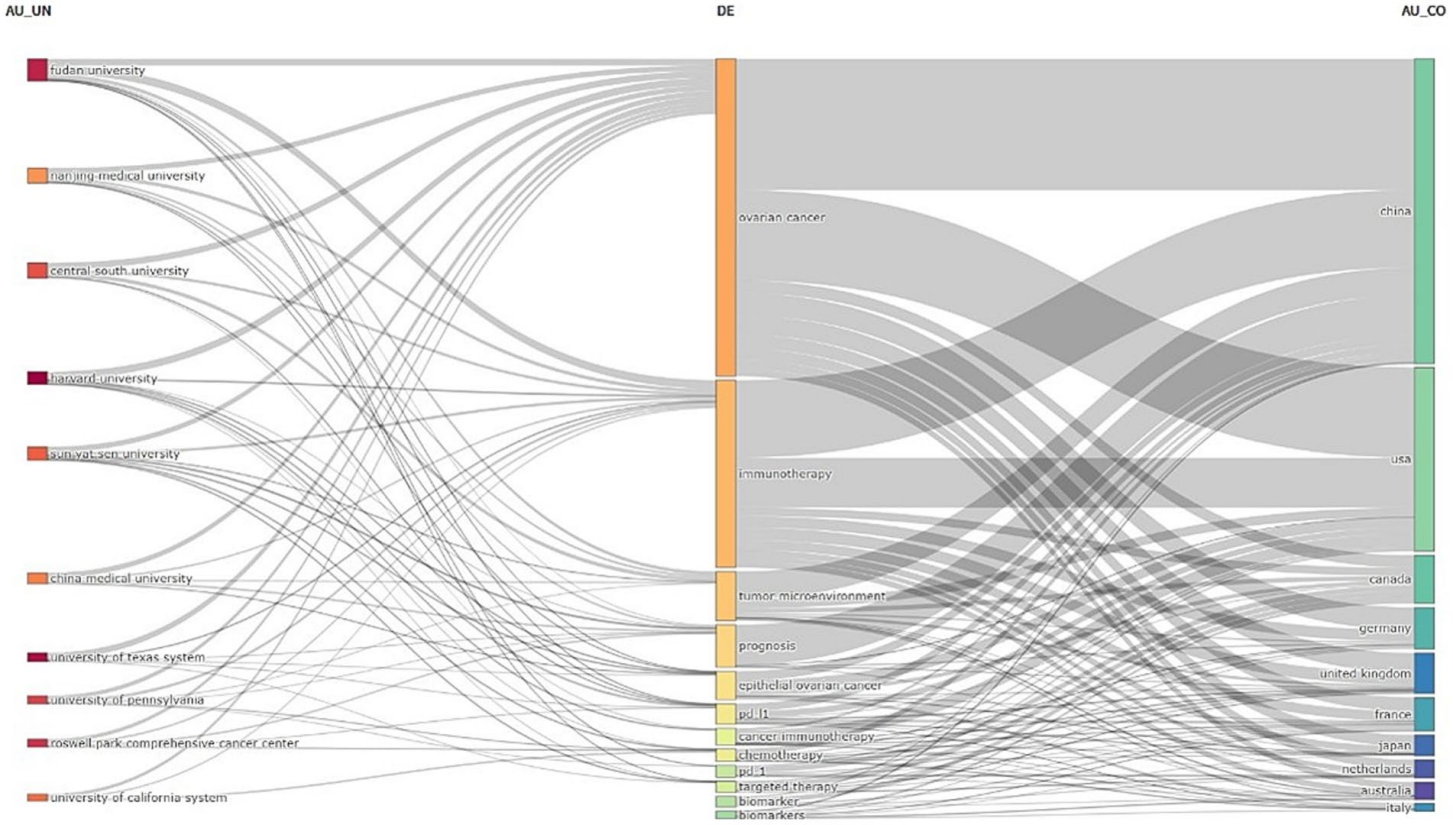
The three-field plot of the keywords plus analysis on ovarian cancer immunotherapy. (Left field: institutions; Middle field: keywords; Right field: countries).

Figure 6 further illustrates the distribution and proportions of core themes among institutions and countries, highlighting the connections between countries, institutions, and keywords in ovarian cancer immunotherapy. Nearly all institutions and countries have contributed to the themes represented by these keywords.

At the institutional level, Fudan University demonstrates a stronger focus on themes related to “tumor microenvironment,” “pd-1,” and “pdl-1.” At the same time, the University of Pennsylvania is centered on “prognosis” and “biomarker.” The University of Texas, in contrast, places greater emphasis on research surrounding “targeted therapy.”

At the national level, both the United States and China have made significant contributions to all of these key themes, particularly in the areas of the keywords: “tumor microenvironment,” “Epithelial ovarian cancer,” “PD - L1,” or “PD – 1,” “Chemotherapy,” “Targeted therapy,” “Biomarker,” “Immune checkpoint inhibitor,” and “Immune microenvironment.” “Immune checkpoint, “Italy shows less interest in themes related to “prognosis,” while Australia and Japan have lower levels of focus on “tumor microenvironment” related themes.

**Figure 7:**
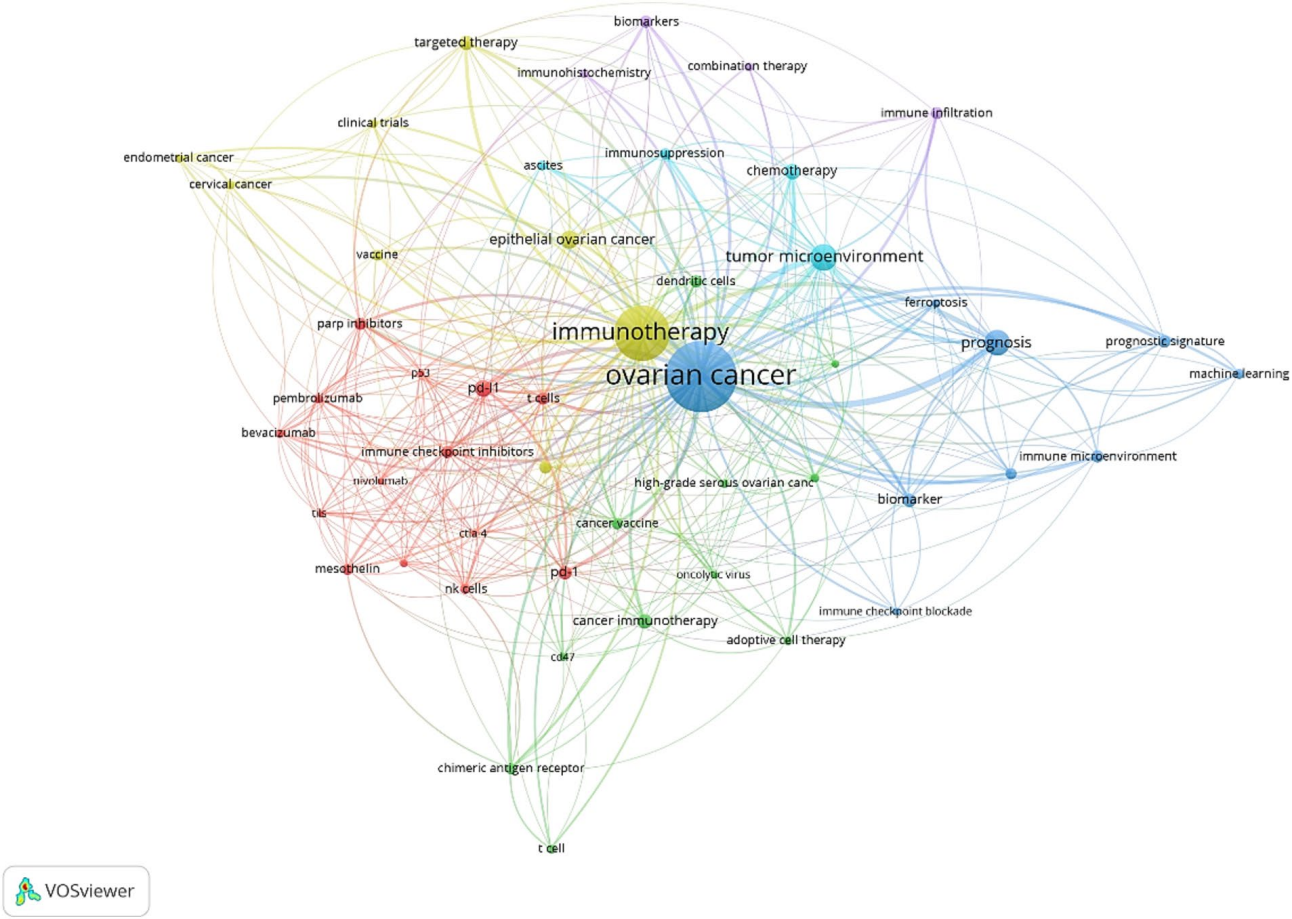
Keyword co-occurrence network.

The co-occurrence analysis of 50 keywords reveals distinct relationship patterns within the field. In this analysis, the size of the dots represents the frequency of keyword usage, while the color indicates keyword clusters. The proximity between dots reflects their correlation’s strength, with keywords with stronger connections grouped in Figure 7.

These 50 keywords are divided into five clusters:

Red Cluster: This group contains 14 keywords and primarily focuses on elements related to the immune system, such as “NK cells,” “CTLA-4,” “mesothelin.” It also includes terms associated with “immunotherapy,” like “pembrolizumab,” and “pembrolizumab.”

Green Cluster: Comprising 11 keywords, this cluster centers on cancer immunotherapy research, a major focus in the field key terms include “adoptive immunotherapy,” “cancer vaccine,” and “chimeric antigen receptor.”

Blue Cluster: The third cluster contains 9 keywords, primarily related to mechanism. It includes terms such as “biomarker,” “immune microenvironment,” “tumor immune microenvironment,” and “machine learning,” emphasizing the pathogenesis exploration of ovarian cancer.

Yellow and purple Clusters: These cluster comprises 12 items, mainly focusing on research approaches for ovarian cancer. Concepts like “immune checkpoints,” “immunotherapy,” “targeted therapy,” “vaccines” and “combination therapy “, reflect recent research trends.

**Figure 8:**
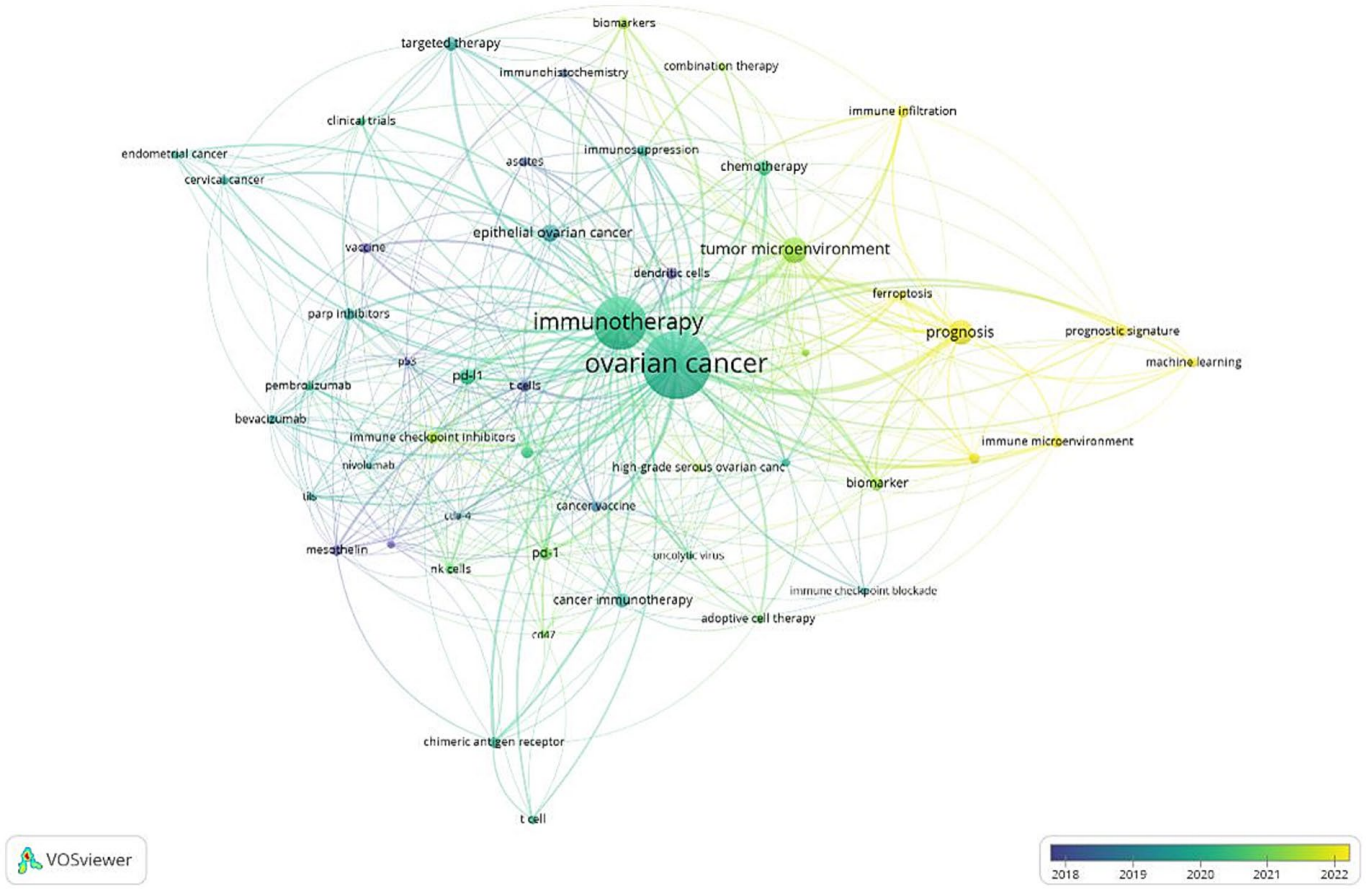
Keyword co-occurrence plus time-overlapping network.

Figure 8 illustrates the time-overlap analysis network of these co-occurring keywords. The color gradient, ranging from dark blue to light blue and from light red to dark red, indicates the average years during which these keywords have garnered the most attention from researchers. Early studies primarily focused on “adoptive cell therapy,” “mesothelin,” and “dendritic cells.” In contrast, more recent research has shifted toward terms such as “prognosis,” “immune infiltration,” “ferroptosis,” and “immune microenvironment,” which have become more prominent in the literature in recent years.

#### Citation burst analysis of keywords

**Figure 9:**
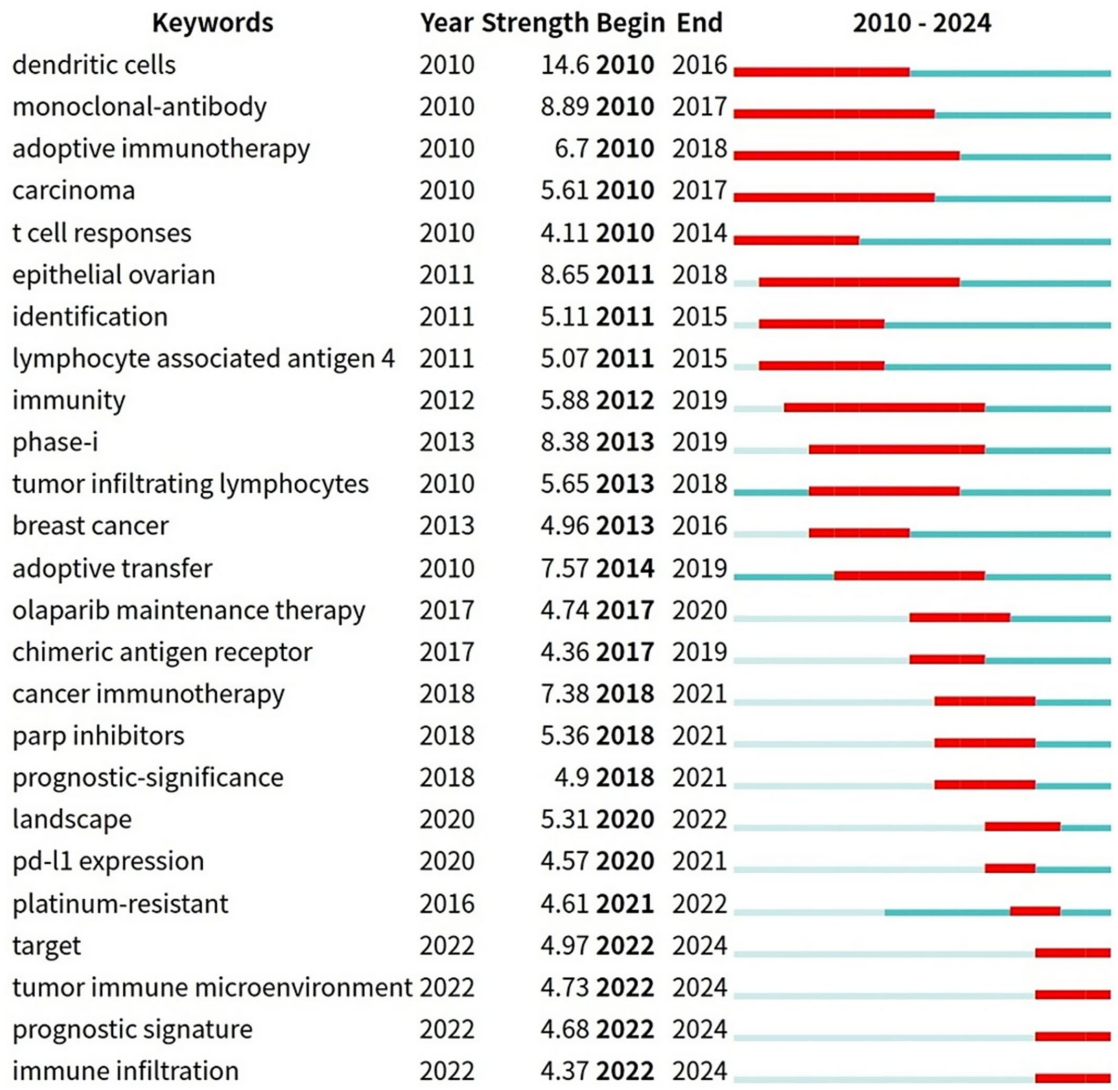
The top 25 keywords with robust citation bursts.

Figure 9 presents the top 25 keywords with the highest citation burst rates. Over time, keywords “adoptive immunotherapy” resistance, with a burst duration from 2010 to 2018, spanning 7 years, “monoclonal-antibody” (2010–2017, 7 years), “carcinoma” (2010–2017, 7 years), “epithelial ovarian” (2011–2018, 7 years), and “immunity” (2012–2019, 7 years) have consistently garnered significant attention. Additionally, terms like “target” (2022–2024), “tumor immune microenvironment” (2022–2024), and “immune infiltration” (2022–2024) have recently gained prominence, highlighting them as key areas of active research, in the past few years and for the near future.

## Discussion

This review employs bibliometric analysis to examine the literature on ovarian cancer immunotherapy published between 2010 and 2025. The first article in this field was published in 2010, which demonstrated that high-grade, drug-resistant ovarian cancer overexpresses epithelial cell adhesion molecule (EpCAM) and is highly sensitive to immunotherapy using MT201, a fully human monoclonal anti-EpCAM antibody (26).

Regarding publication volume, the trend can be divided into two phases: slow growth and rapid growth. From 2010 to 2019, ovarian cancer immunotherapy experienced a slow growth, with less than 1,000 papers published yearly. However, from 2020 to 2024, the field has made significant progress and entered a rapid growth stage, with more than 200 papers published yearly. This shows that the research on ovarian cancer immunotherapy is in the prosperity stage. Several factors may lead to this phenomenon. First, ovarian cancer is characterized by tumor heterogeneity and immune evasion mechanisms, leading to poor treatment outcomes. This urgently requires innovative treatment options, such as immunotherapy. Ovarian cancer exhibits a range of immunosuppressive mechanisms, such as overexpression of immune checkpoint molecules such as PD-1/PD-L1 (27), which suppress the immune system and help the cancer resist conventional therapies. Immunotherapy aims to reactivate the immune system to fight the proliferation of tumor cells.

Secondly, the study of the tumor microenvironment (TME) of ovarian cancer (28) plays a key role. TME is critical in tumor progression and immune resistance. With in-depth research on TME, new targeted therapies continue to emerge, including immune checkpoint inhibitors (29) and CAR-T cell therapy (30), bringing new hope to patients.

The top 10 contributing countries account for 81.8% of total publications in this field. China leads the world in the number of publications and plays a key role in international cooperation. As shown in the figure, in cutting-edge research on ovarian cancer immunotherapy, the United States leads the way with breakthrough research results and major advances. It is at the forefront of this field. Despite China’s rapid economic growth, heavy investment in healthcare is a key factor in its success. Future economic support and international collaborations are expected to further drive growth in this sector.

The United States ranks second in publishing articles on immunotherapy research for ovarian cancer, with the top five institutions in the United States benefiting from its advanced healthcare system and strong research infrastructure. On the other hand, China leads in this field, with the largest number of articles published in the world and five Chinese universities in the global top 10. This highlights China’s growing prominence in ovarian cancer immunotherapy research and reflects its growing investment in medical research, especially cancer treatment innovation.

In terms of recent activity, two Chinese universities, Fudan University and Central South University, have shown the most active performance in the field of ovarian cancer immunotherapy research in recent years. Research competitiveness will be enhanced through international collaborations, suggesting that seeking broad cooperation between institutions is crucial, particularly in situations where economic or resource constraints exist.

Regarding journal impact, impact factors and Journal Citation Reports (JCR) are effective indicators for assessing journal influence. Among the top 10 journals, 80% are ranked in JCR Q1. However, only two journals in the top 10 have published more than 50 articles on ovarian cancer immunotherapy. These include Cancers and Frontiers in Immunology, which have the highest publication volumes. Core journals often bear the responsibility of publishing key research in their respective fields, and these top journals can be recommended to researchers for submission. Despite China’s significant contributions to this field, none of the top 10 journals are published by Asian publishers. This indicates that China can establish journals with international influence.

One of the goals of this study is to identify the scientific hotspots in ovarian cancer immunotherapy, which can be analyzed through publications, references, and keywords. The number of citations a publication receives can serve as one of its indicators of impact (31). Frequently cited publications reflect core themes in the field, helping to identify research hotspots. Overall, the 10 most cited papers focus on several key areas: immune checkpoint inhibitors (such as PD-1/PD-L1 inhibitors), cellular therapies in immunotherapy, and the tumor immune microenvironment and immune evasion mechanisms.

In 2014, Webb et al. (32) and colleagues published a study in CLIN CANCER RES exploring the potential role of CD103-positive tumor-infiltrating lymphocytes (TILs) in immune surveillance in ovarian cancer. This study shows that detecting CD103 positive T cells in tumors may help predict the ending of patients with high -risk plasma ovarian cancer. Subsequently, in 2019, Barkal et al. (33) and his colleagues published a paper that revealed the key role of CD24-Siglec-10 in tumor immunization. They recommend that this way can restore the anti-tumor activity of macrophages, thereby enhancing the ability of the immune system to eliminate tumors. These two studies jointly provide important insights on tumor immune microenvironment and immune escape mechanism, to provide valuable views on the interaction between tumors and immune systems. These findings also guide new strategies for the development of ovarian cancer immunotherapy.

In 2015, Hamanishi et al. (34) and colleagues studied the safety and antitumor activity of the anti-PD-1 antibody Nivolumab in patients with platinum-resistant cancers. This study explores the application of immune checkpoint inhibitors in ovarian cancer. In the same year, Peng et al. (35) and other scholars studied how chemotherapy promotes the expression of PD-L1 through the NF-κB pathway, thereby creating an immunosuppressive tumor microenvironment and its impact on the effectiveness of immunotherapy.

Cell-based therapy is one of the key strategies to enhance the efficacy of immunotherapy. In 2015, Koneru et al. (36) and her team investigated using IL-12-secreting, tumor-targeted CAR-T cells in treating ovarian cancer. In 2016, Wang et al. (28) and colleagues explored how effector T cells can overcome chemotherapy resistance mediated by the tumor stroma. These studies provide an important foundation for the further application of cell-based therapies in cancer treatment.

The most cited article in this field was published in 2019 by Lheureux et al. (21). This article explores the importance of genetic mutations, such as BRCA1/2 mutations, in ovarian cancer and highlights recent advances in therapeutic strategies based on tumor molecular signatures. Although the effectiveness of immune checkpoint inhibitors in ovarian cancer remains uncertain, genomic profiling and immune cell profiling may help identify patients who may respond to immunotherapy. Precision medicine plays a key role in this process by screening biomarkers, which not only help predict response to immunotherapy but also help identify ovarian cancer patients who are suitable for such treatment, thus helping to personalize treatment development. In 2018, Cortez et al. (37) and colleagues examined a variety of treatments, ranging from traditional to emerging therapies, in “Advances in Ovarian Cancer Treatment.” This summary focuses on the use of immunotherapy point inhibitors in ovarian cancer and combined therapy strategies, such as the combination of immunotherapy and chemotherapy or targeted therapy to improve the treatment effect. These two articles outline the total use of immunotherapy and other treatment methods in the treatment of ovarian cancer.

In 2018, Tanyi et al. (38) and his colleagues conducted a study to apply personalized cancer vaccine to ovarian cancer, focusing on the way to resist tumor by activating specific T cell immune reactions. In 2020, Lindsay Kurokis’ team (39) published a systematic review that comprehensively summarized various ovarian cancer treatment strategies, including immunotherapy point inhibitors, tumor vaccines, and immunotherapy. This review highlights further optimization of combination therapy and immunotherapy approaches.

Keywords represent the core theme of research and their frequency reflects the influence of specific areas in one field. In the research on ovarian cancer immunotherapy the most used keywords are closely related to immune aged ingredients such as expression T cells regulating T cells and dendritic cells. These keywords highlight the central theme of research. In recent years the analysis of keywords has identified the most commonly used terms particularly “tumor microenvironment,” “pd-l1/ pd-1,” “targeted therapy,” and “immune checkpoint inhibitors,” which have garnered significant attention in the academic community.

Citation burst analysis is a method provided by CiteSpace for identifying references and keywords that have experienced significant changes over a specific period. Citation intensity reflects the extent to which a reference or keyword is widely discussed and frequently cited, which is an important indicator of research hotspots and influence. The duration of a citation burst indicates how long a paper has been cited and whether it has attracted attention recently. In this study, keywords such as “dendritic cells,” “monoclonal-antibody,” and “adoptive immunotherapy” experienced a significant citation explosion. Since 2025, the number of relevant papers has increased significantly, among which 8 highly cited papers stand out. This trend is still rising.

Keyword clustering analysis reveals two key biological themes driving ovarian cancer immunotherapy research. First immune checkpoint-related keywords (e.g., PD-1/PD-L1 CTLA-4) highlight tumor immune evasion mechanisms as seen in Peng et al. (35) who found that nivolumab upregulates PD-L1 via the NF-κB pathway to create an immunosuppressive microenvironment. Similarly T-cell-related terms (e.g., CAR-T TILs) emphasize T-cell infiltration’s prognostic value—Webb et al. (32) linked CD103 + TILs to improved survival—Justifying immunotherapy strategies to enhance T-cell activity. Emerging research also identifies cell death mechanisms (e.g., pyroptosis) as therapeutic targets with Wang et al. (28) proposing that pyroptosis could reprogram the tumor microenvironment to amplify anti-tumor responses.

However, translating these findings into clinical practice faces challenges. Immune checkpoint inhibitors show limited efficacy, with Hamanishi et al. (34) reporting a 15% ORR for nivolumab in platinum-resistant patients, underscoring monotherapy’s limitations. Combination therapies, such as personalized vaccines with PD-1 inhibitors (38), show promise but require robust biomarker development (e.g., TMB, PD-L1) for patient stratification. Resistance mechanisms, including tumor heterogeneity and TAM-mediated immune suppression (4), remain significant hurdles. Future research should adopt a mechanism-driven, biomarker-guided approach and strengthen international collaborations to overcome these challenges and maximize clinical impact.

### Key recent publications

Pyroptosis and the Tumor Immune Microenvironment: A New Battlefield in Ovarian Cancer Treatment (40). This article explores the role of pyroptosis in the tumor immune microenvironment of ovarian cancer, highlighting its profound impact on tumor growth, immune regulation, and therapeutic response. Research shows that induction of apoptosis or regulation of related signaling pathways may provide new targets and strategies for the treatment of ovarian cancer, thus opening more effective therapeutic pathways.

Trabectedin and Lurbinectedin Modulate the Interplay Between Cells in the Tumor Microenvironment—Progresses in Their Use in Combined Cancer Therapy (41). This article discusses the impact of chemotherapy drugs TrabeCTEDIN and Rubinadine on the micro-environment of tumor, focusing on their influence on tumor -related macrophages (TAMS). In addition, this article reviews the progress of combining these drugs with other cancer treatment.

Limitations and Potential of Immunotherapy in Ovarian Cancer (4). This study provides a comprehensive analysis of the limitations of immunotherapy in ovarian cancer, examining how the immunosuppressive characteristics and low mutational burden of the tumor microenvironment can lead to suboptimal treatment outcomes. Furthermore, the study suggests potential improvement strategies, including combined therapies and targeting the tumor microenvironment to improve treatment efficacy.

Comprehensive analysis of mitophagy-related genes and mitophagy-related lncRNAs in ovarian cancer patients (42). This article comprehensively analyzes mitophagy-related genes and lncRNAs in ovarian cancer to reveal their regulatory mechanisms and their impact on tumor progression. These molecules have been proposed as potential biomarkers and therapeutic targets, providing new ideas for personalized treatment.

Cyclooxygenase-2 Blockade Is Crucial to Restore Natural Killer Cell Activity Before Anti-CTLA-4 Therapy Against High-Grade Serous Ovarian Cancer (43). This study shows that blocking cyclooxygenase-2 (COX-2) restores natural killer (NK) cell function and enhances the efficacy of anti-CTLA-4 immunotherapy in high-grade plasma ovarian cancer. This study suggests that COX-2 inhibitors may be a potential combination treatment strategy to improve treatment outcomes.

### Emerging research directions

Immunotherapy research for ovarian cancer continues to evolve, particularly in understanding the tumor microenvironment, immunotherapy targets, and predicting treatment response. Although immune checkpoint inhibitors (ICIs) and adoptive immunotherapies (e.g., CAR-T cell therapy) show promise in the treatment of other cancers, their efficacy in ovarian cancer remains limited due to factors such as the immunosuppressive tumor microenvironment, low mutational load, and pyroptotic mechanisms. Studies have shown that tumor burden indicators, such as tumor mutation load, immune infiltration signatures, and cell death types (such as pyroptosis and autophagy), can help predict response to immunotherapy.

Furthermore, platinum resistance remains a major challenge in ovarian cancer treatment. This study focuses on developing strategies to overcome these challenges, specifically targeting cyclooxygenase-2 (COX-2) and tumor-associated macrophages (TAMs), which hold promise for restoring immune cell function and enhancing the effectiveness of immunotherapy. For patients with platinum-resistant ovarian cancer, exploring open-label strategies combined with immunotherapy has become a focus.

All in all, the future of ovarian cancer immunotherapy will largely depends on the in -depth understanding of tumor microenvironment, integration of cell death mechanism, and in -depth understanding of drug resistance. Combining personalized therapy and combination of combination therapy will be the key to improving the effectiveness of immunotherapy.

### Challenges and future directions

Despite the potential of immunotherapy in ovarian cancer, its clinical translation faces significant hurdles. Tumor heterogeneity and the “cold tumor” phenotype limit the efficacy of monotherapies by enabling immune evasion, while the relationship between platinum resistance and immunosuppressive tumor microenvironments remains poorly understood. These factors collectively constrain therapeutic response rates in clinical settings.

To address these challenges, future research should focus these priorities: developing predictive biomarkers such as T - cell clonality and spatial transcriptomics to enable patient stratification; optimizing combination therapies, including integrating immunotherapy with epigenetic modulators to overcome resistance mechanisms; and utilizing humanized preclinical models that accurately recapitulate clinical resistance patterns for more effective translational research. These strategies aim to enhance clinical response rates and accelerate therapeutic advancements in ovarian cancer treatment.

### Limitations of this study

This review has several limitations. First, bibliometric analysis, data collection and processing heavily rely on software tools and cannot completely replace systematic literature searches. However, this method helps extract comprehensive analytical results from large datasets. Second, the study only included English-language publications from the Web of Science Core Collection (WoSCC) database, which may have excluded some valuable non-English studies. Nevertheless, the WoSCC database covers the most relevant research, and this omission is unlikely to significantly impact the overall trends observed. Third, citation impact has a lag effect, and recent high-quality studies may not yet fully reflect their academic influence, which should be updated in future research.

Despite these limitations, this study offers valuable insights into the development trends, research hotspots, and cutting-edge directions in the tumor burden and immunotherapy field.

## Conclusion

Ovarian cancer immunotherapy research will increasingly focus on tumor microenvironment modulation, exploration of cell death mechanisms, overcoming resistance, innovative combination therapies, and the development of biomarkers to predict treatment responses. These advancements will be essential for the future of ovarian cancer immunotherapy.

## Footnotes

^1^ https://bibliometric.com/
