# Supplementary material for: Visual analysis of ovarian cancer immunotherapy: a bibliometric analysis from 2010 to 2025
Source: Front Med (Lausanne). 2025 Jul 11;12:1573512. doi: 10.3389/fmed.2025.1573512 (PMC12291683; doi:10.3389/fmed.2025.1573512)
Supplement: Supplementary file 1 [file Table_1.docx]

Supplementary Material

**Visual Analysis of Ovarian Cancer Immunotherapy: A Bibliometric Analysis From 2010 to 2025**

Ying-jie Zhang, Yan-yan Chen, Chun-ru Chen, Xiao-hua Cheng, Ya-nan Peng, Juan Wang， Fu-xia Li，Wen-ting LI

*** Correspondence: Wen-ting LI**: [124873340@qq.com](http://124873340@qq.com)

# Supplementary Figures and Tables

## Supplementary Figures


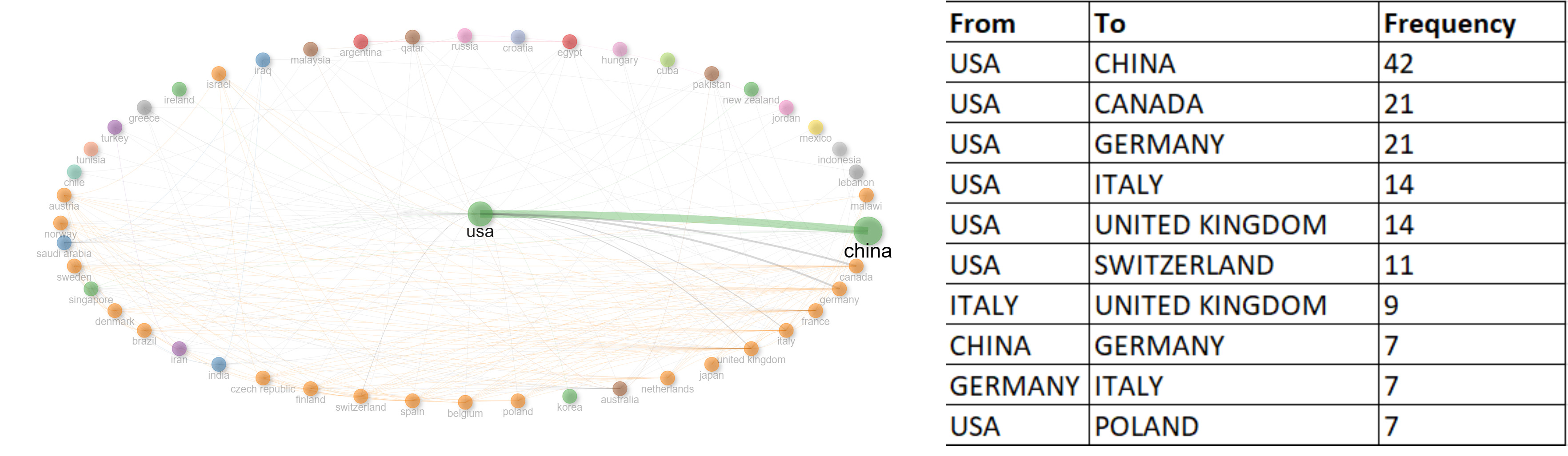


**Supplementary Figure 1.** International collaboration and high frequency collaboration countries in the field of ovarian Cancer Immunotherapy.


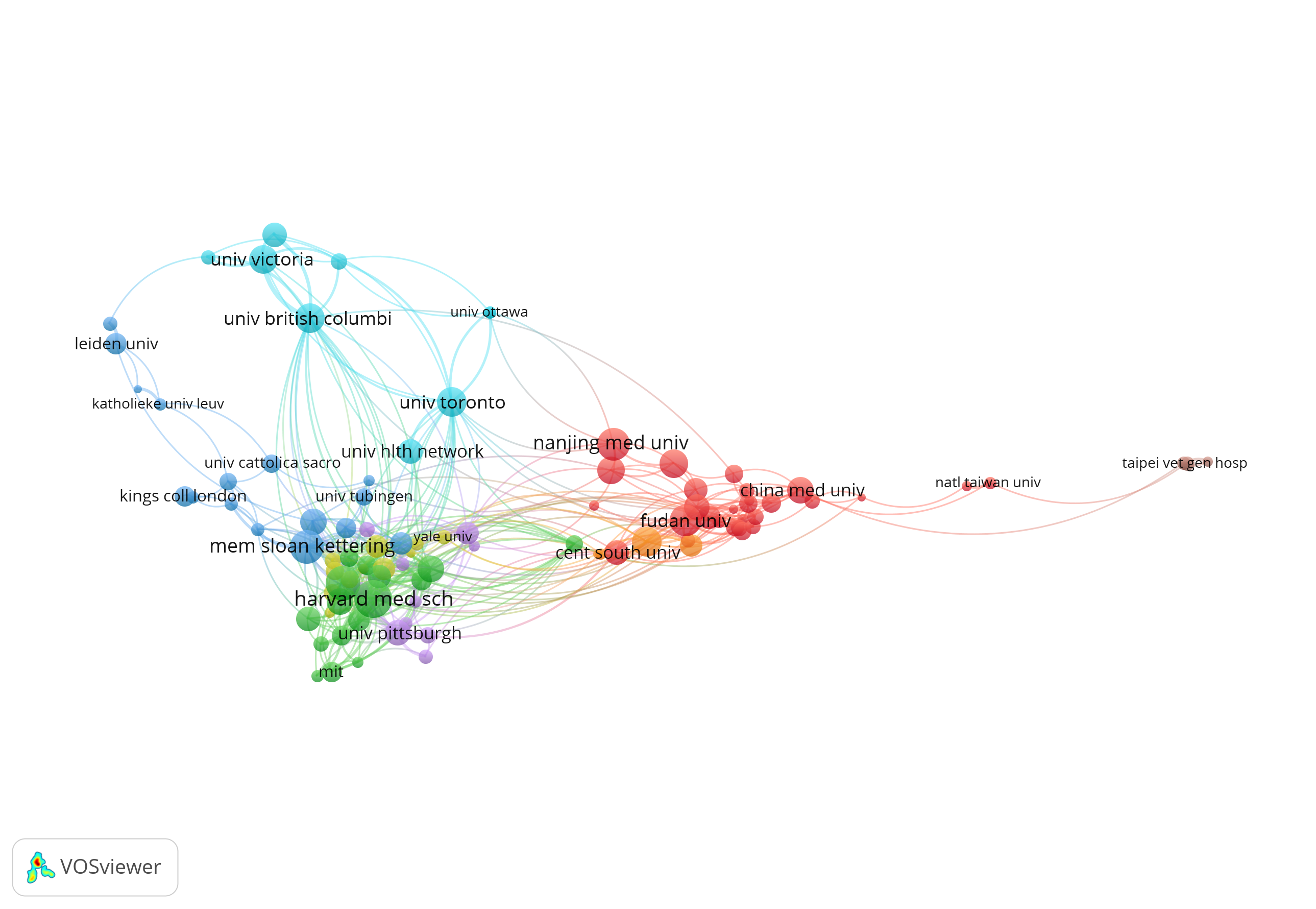
**Supplementary Figure 2A.** Clustering network for institutional co-authorship analysis


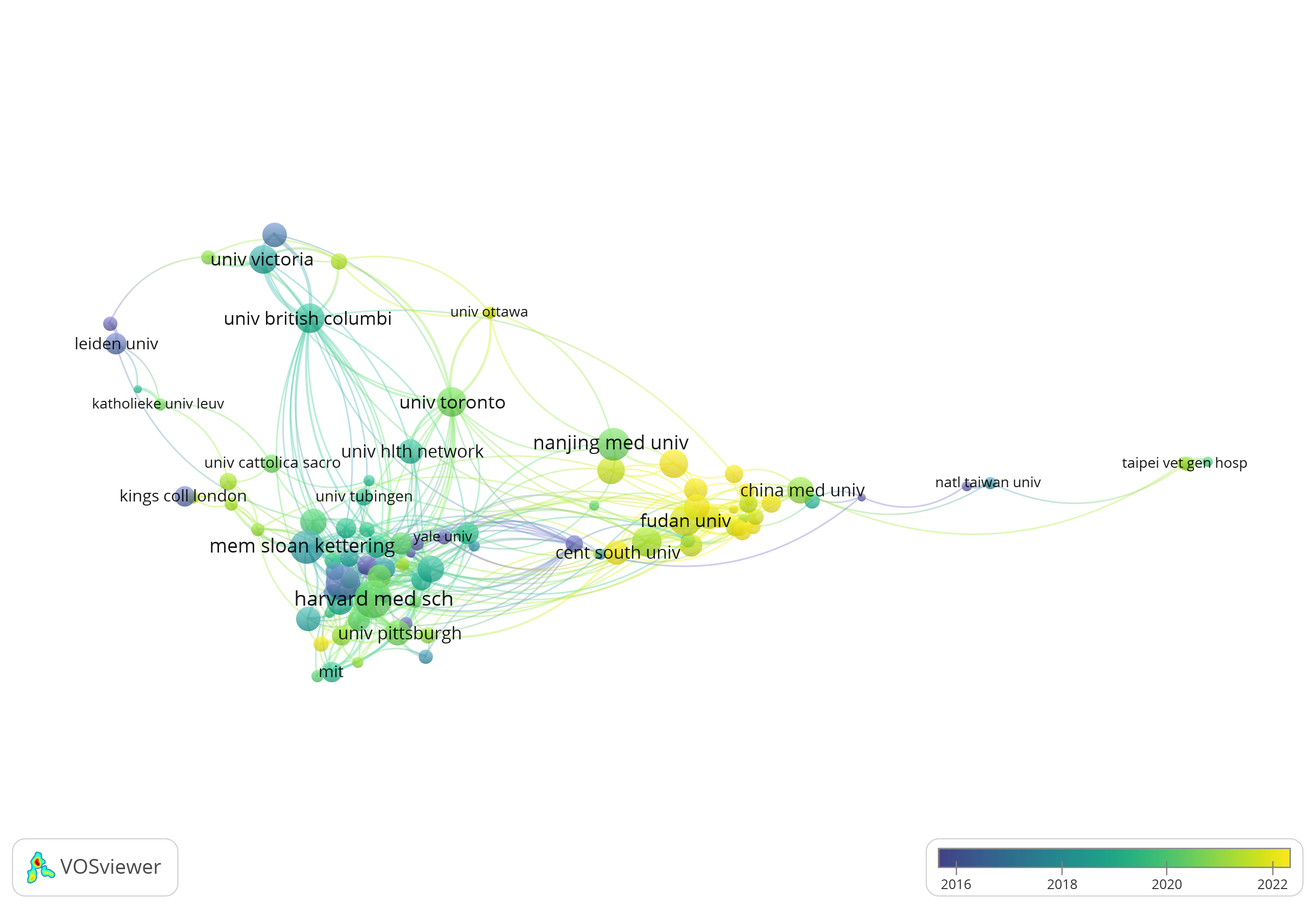


**Supplementary Figure 2B.** Time-overlapping visualization for institutional co-authorship analysis


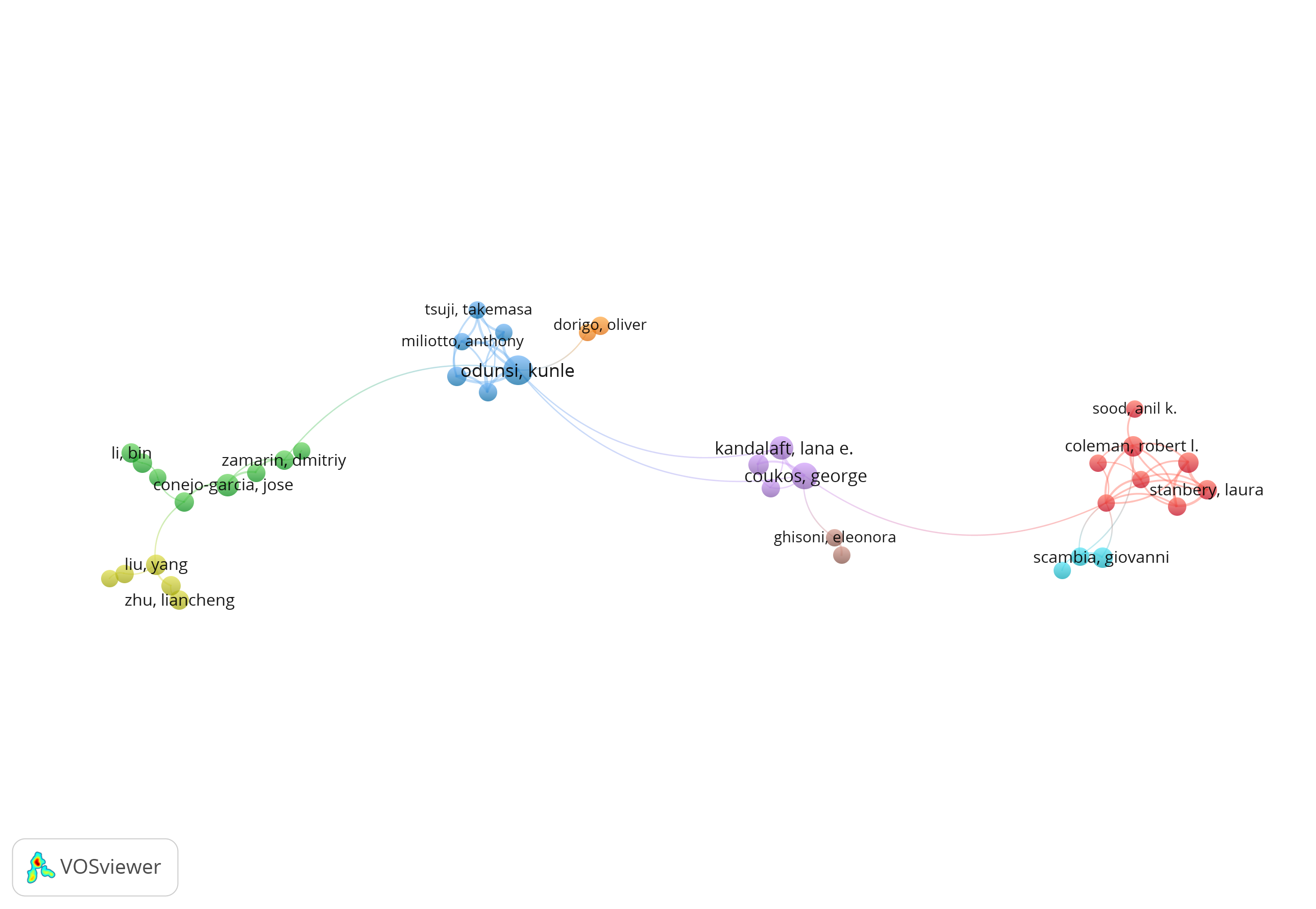


**Supplementary Figure 3A.** Clustering network for author co-authorship analysis


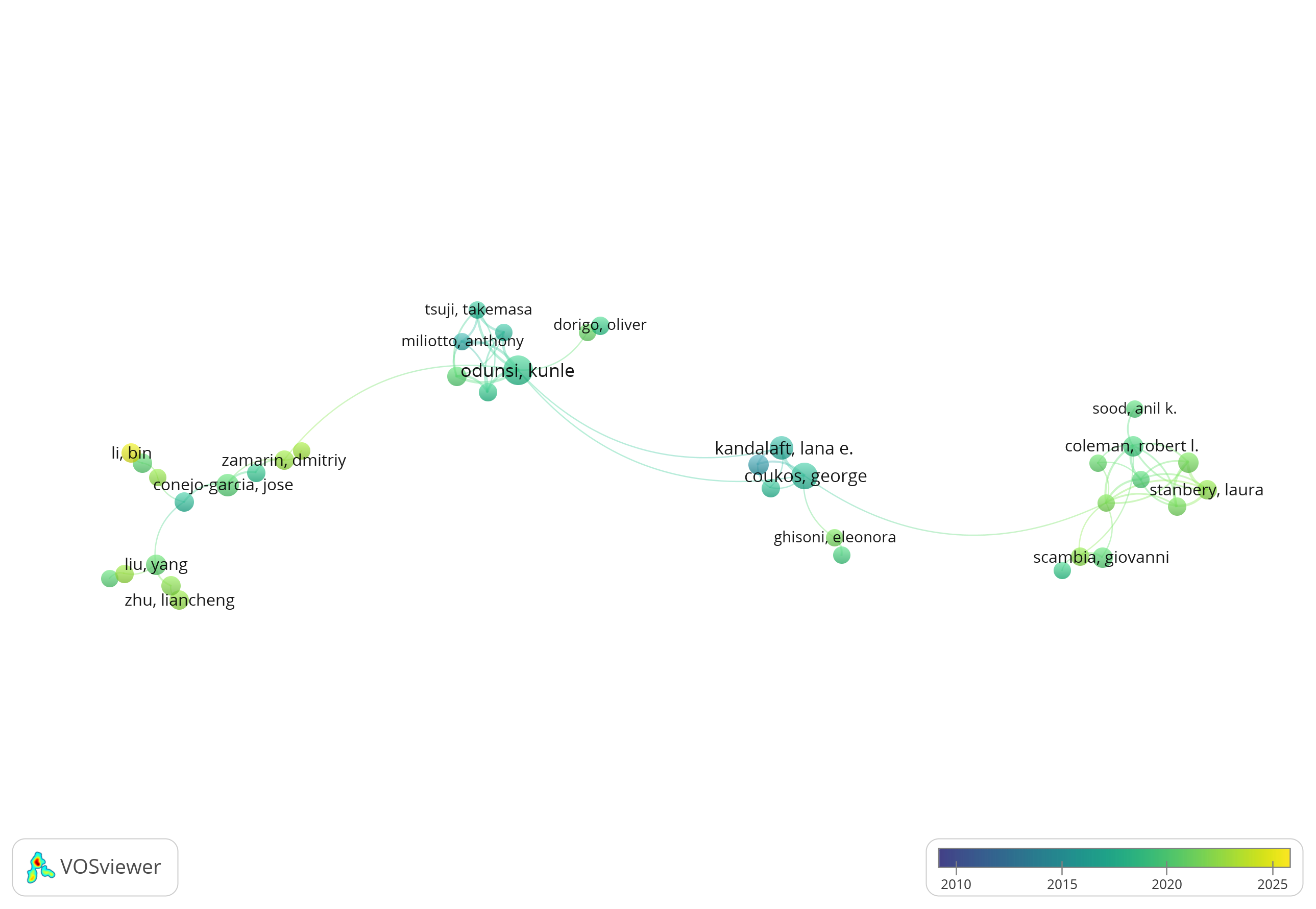


**Supplementary Figure 3B.** time-overlapping visualization for author co-authorship analysis


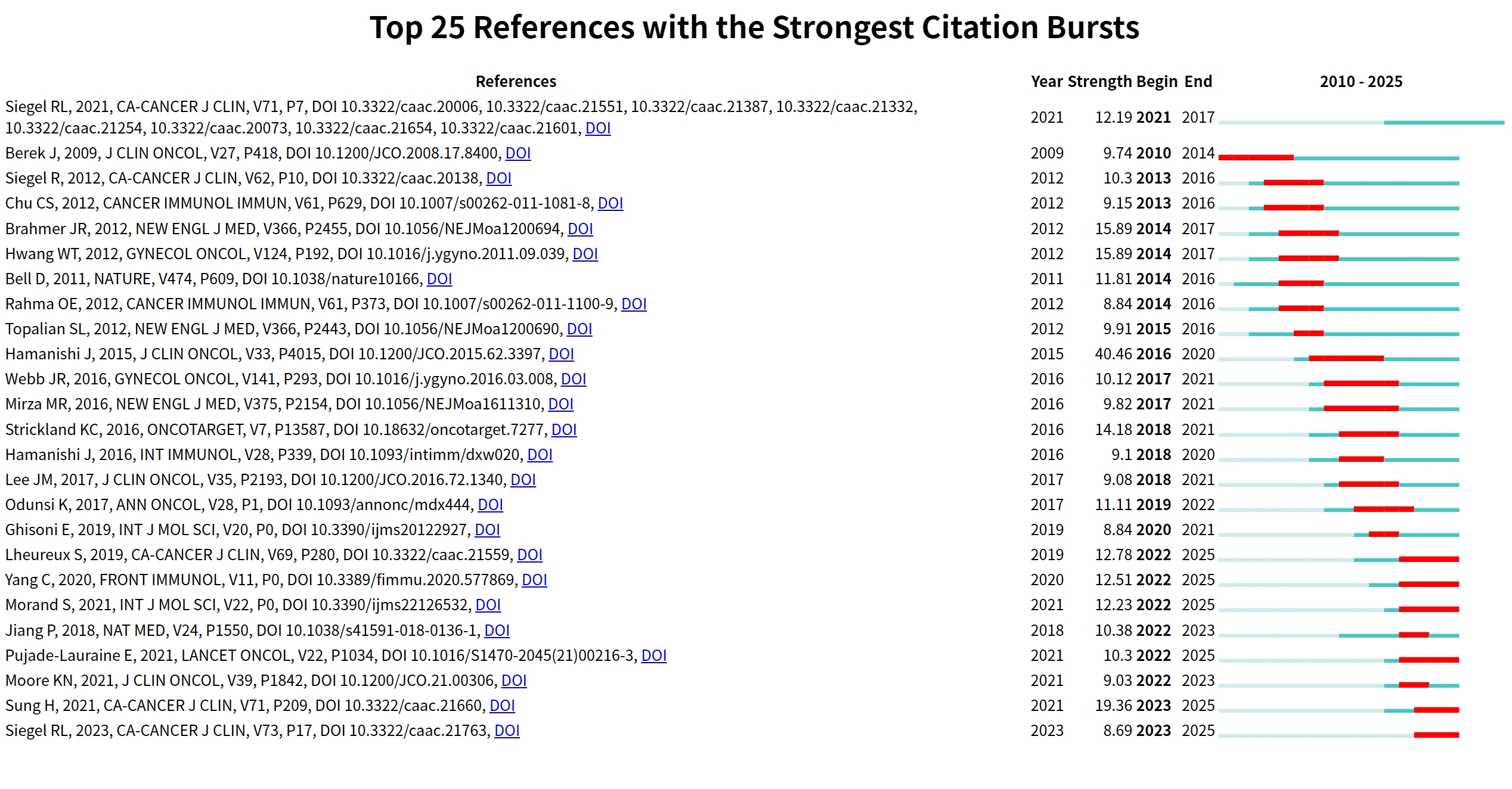


**Supplementary Figure 4.** Top 25 cited references with the strongest citation bursts on ovarian Cancer Immunotherapy

## Supplementary Tables

**Supplementary Table 1**. The top 10 cited publications

| **Rank** | **Title** | **Year, Journal** | **First author** | **Total Citations** | **TC per Year** |  |
| --- | --- | --- | --- | --- | --- | --- |
| 1 | Epithelial ovarian cancer: Evolution of management in the era of precision medicine | 2019, CA-CANCER J CLIN | Stephanie Lheureux | 854 | 122 |  |
| 2 | Safety and Antitumor Activity of Anti–PD-1 Antibody, Nivolumab, in Patients With Platinum-Resistant Ovarian Cancer | 2015, J CLIN ONCOL | Junzo Hamanishi | 820 | 74.55 |  |
| 3 | CD24 signalling through macrophage Siglec-10 is a target for cancer immunotherapy | 2019, NATURE | Amira A. Barkal | 694 | 99.14 |  |
| 4 | Chemotherapy Induces Programmed Cell Death-Ligand 1 Overexpression via the Nuclear Factor-κB to Foster an Immunosuppressive Tumor | 2015, CANCER RES | Jin Peng | 416 | 37.82 |  |
| 5 | Treatment of epithelial ovarian cancer | 2020, BMJ-BRIT MED J | Lindsay Kuroki | 404 | 67.33 |  |
| 6 | Advances in ovarian cancer therapy | 2018, CANCER CHEMOTH PHARM | Alexander J. Cortez | 383 | 47.88 |  |
|  |  |  |  |  |  |  |
| 7 | IL-12 secreting tumor-targeted chimeric antigen receptor T cells eradicate ovarian tumors in vivo | 2015, ONCOIMMUNOLOGY | Mythili Koneru | 317 | 28.82 |  |
| 8 | Tumor-Infiltrating Lymphocytes Expressing the Tissue Resident Memory Marker CD103 Are Associated with Increased Survival in High-Grade Serous Ovarian Cancer | 2014, CLIN CANCER RES | John R. Webb | 311 | 25.92 |  |
| 9 | Effector T Cells Abrogate Stroma-Mediated Chemoresistance in Ovarian Cancer | 2016, CELL | Weimin Wang | 298 | 29.8 |  |
| 10 | Personalized cancer vaccine effectively mobilizes anti-tumor T cell immunity in ovarian cance both innate and adaptive immunity | 2018, SCI TRANSL MED | Janos L. Tanyi | 292 | 36.5 |  |

**Supplementary Table 2**. Citing from national publications

| **Country** | **TC** | **Average Article Citations** |
| --- | --- | --- |
| USA | 12046 | 34 |
| CHINA | 5149 | 8.9 |
| CANADA | 2749 | 53.9 |
| JAPAN | 2007 | 64.7 |
| GERMANY | 1025 | 30.1 |
| ITALY | 904 | 26.6 |
| UNITED KINGDOM | 902 | 26.5 |
| POLAND | 710 | 41.8 |
| NETHERLANDS | 704 | 26.1 |
| SWITZERLAND | 613 | 43.8 |

**Supplemental Digital Content 1**. high frequency collaboration countries in the field of ovarian Cancer Immunotherapy.
